# In the digital world of the EMR, Open Notes change everything

**DOI:** 10.1186/s13584-015-0012-0

**Published:** 2015-03-31

**Authors:** Debra L Roter

**Affiliations:** Department of Health, Behavior, and Society, Johns Hopkins Bloomberg School of Public Health, 624 N. Broadway, Baltimore, MD 21205 USA

**Keywords:** E-heath, Electronic Medical Records (EMR), Health information technology (HIT), Patient-centered communication, Patient engagement, Patient activation

## Abstract

EMR use during medical visits has been found to distract physicians and negatively influence their ability to deliver patient-centered care. In this issue, Assis-Hassid and colleagues propose a redress of this effect by creating a 23-item inventory of communication-related best practices regarding EMR use during medical visits with relevance for medical student training and enhanced clinical practice. This paper and recent initiatives to share physicians’ EMR notes with patients through secure portals raise questions regarding the future role of the EMR and its implication for the patient-physician relationship.

The purpose of this commentary is to provide a perspective on the EMR as part of the rapidly evolving digital environment and to discuss how the growing movement to provide patients full access to their EMR can act as a catalyst for the forging of a new model of patient-physician collaboration and partnership.

The ubiquitous presence of the Electronic Medical Record (EMR) in the exam room has shaped medical practice in largely beneficial ways. However, it has also raised concerns regarding unanticipated consequences for patient-provider communication during medical visits. As noted by Assis-Hassid and colleagues in their article in the current issue of the Israeli Journal of Health Policy Research [1], studies in Israel and elsewhere have suggested that EMR use may have a negative effect on the doctor-patient relationship by diverting physician attention away from the patient to the computer screen.

In an attempt to redress this effect, the authors conducted a comprehensive literature review to generate a list of computer-related communication practices that was validated through an online survey of medical students and faculty and a consensus meeting of primary care physicians with a special interest in EMR implementation and communication. These processes produced a list of 23 items that reflect an inventory of communication-related best practices regarding EMR use during medical visits. If I have any reservation in regard to the contribution of the authors, it is not because I question the importance of the items they have identified but because I question the insularity of the items from the wide-ranging digital context within which medical practice now firmly lives.

As noted by Jonathan Weiner in a commentary to this journal several years ago, the e-health revolution is upon us and almost all patient/provider contact in the future including those occurring before, during and after traditional face-to face medical visits will be mediated by health information technology (HIT) [2]. This technology goes well beyond the EMR to information systems that facilitate patient contact not only with clinicians but also with the institutions within which care is delivered and health information safeguarded. While much of HIT can be seen as providing functional advances for the accomplishment of administrative and documentation tasks, some technology may indeed be revolutionary with the potential to positively re-shape the nature of the patient-physician relationship. It is within this context that EMR of the future has an important role to play.

The traditional EMR that is physician authored, accessed and shielded from patients has been described as a third party interloper in the medical visit that distracts physicians from patients and their ability to deliver patient-centered care. It can, however, be something different; it can become a vehicle of patient engagement and empowerment. The purpose of this commentary is to provide a perspective on the EMR as part of the wider digital medical environment and to discuss how the growing movement to provide patients full access to their EMR can act as a catalyst for the forging of a new model of patient-physician collaboration and partnership.

There have been small studies, some as long as 25 years ago, that have provided patient access to medical records, but none of these have been on the scale pioneered by Delbanco and colleagues in their Open Notes initiative [3]. More than 100 primary care physicians and 20,000 of their patients in three major American health care systems were enrolled in a one-year demonstration project during which patients were given access to their medical records through secured health system portals. The investigators were interested in exploring three questions: [1] would patients read their notes and will those who do read them report greater engagement in their care and management of their health?; [2] would patients access their medical notes in a way that does not negatively impact physicians’ practice patterns and productivity?; and, [3] would patients and physicians want to continue Open Notes in the future?

The answer was an unequivocal YES to all of these questions.

Open Notes use was high, ranging from 47% to 92% across systems. Even more impressive was that over 75% of patients completing a post-program survey reported that Open Notes helped them feel more in control of their care and increased medication adherence. Moreover, 99% of patients wanted to continue Open Notes in the future. Physicians were initially reluctant participants in the program, fearing that allowing patient access to their notes could jeopardize their relationship and disrupt their workflow. These fears were largely unfounded. Patient report of confusion, worry, or offense was infrequent and physicians reported that opening their notes to patients strengthened their relationships by enhancing trust, transparency, communication and shared decision making. It also was reported that some patients seemed to be activated and empowered by the shared notes and it may have led to improved patient satisfaction, safety, and patient education. Fears of logistical problems were also unsubstantiated and patient access to notes created little disruption in physician practice patterns. The volume of electronic messages from patients did not change as a result of the program and few doctors reported longer visits or taking much time to address patients’ questions outside of visits. At the end of the demonstration period, no doctor elected to stop participation in Open Notes.

When asked to imagine what a future Open Notes process might look like, some two-thirds of patients believed that they should be able to comment on notes in their medical record and one-third believed that they should have the right to approve their doctors’ notes. In effect, these patients want to go beyond access to co-authorship of their medical records. One-third of their doctors agreed that patients should be able to add notes to their medical record but few went so far as to agree that patients should have the right to approve physician notes. Indeed the proposition of co-authorship raises logistic and legal issues but it also presents exciting possibilities for meaningful collaboration and active patient engagement in care [3].

A minority of patients, some 19% of those who had read a chart note, reported mentioning it to their doctor. It is here that the potential contribution of Assis-Hassid and colleagues’ EMR recommendations are particularly relevant. One can imagine how much more powerful Open Notes could be if EMR documentation during medical visits was regarded by both patients and physicians as an opportunity to facilitate patient collaboration and engagement rather than a distraction. Several of the skills listed in the EMR recommendations are designed to engage patients in their care but far more must be done if the EMR is to be recast as a co-constructed product reflecting a working partnership between the patient and physician.

Recent updates on Open Notes by its authors [4,5] report that not only have the three health systems that participated in the demonstration project continued the initiative but many others, including the Veterans Administration, have opened physician notes to their patients. At the end of 2014, it was estimated that some five million US patients have online access to their physician notes [5]. This number is growing exponentially and Open Notes is approaching the tipping point.

The potential of Open Notes to disrupt the status quo and introduce new possibilities by which the EMR can revolutionize the relationship between patients and physicians is a remarkable reflection of the transformative power of HIT. While realization of a fully digitized medical environment, including widespread implementation of the EMR has been slow, far slower in the US than in Israel and elsewhere, the momentum of Open Notes is a game changer. The need for research and training in the new medicine of collaborative partnerships is greater than ever; change is coming fast and communication is at its heart.
